# Prevalence of Transmitted HIV drug resistance in antiretroviral treatment naïve newly diagnosed individuals in China

**DOI:** 10.1038/s41598-018-29202-2

**Published:** 2018-08-16

**Authors:** Shuai Zhao, Yi Feng, Jing Hu, Yang Li, Zhongbao Zuo, Jing Yan, Jing Zhang, Pi Cao, Wei Xu, Fan Li, Yuxueyun Li, Lingjie Liao, Yuhua Ruan, Yiming Shao, Hui Xing

**Affiliations:** State Key Laboratory of Infectious Disease Prevention and Control, National Center for AIDS/STD Control and Prevention, Chinese Center for Disease Control and Prevention, Collaborative Innovation Center for Diagnosis and Treatment of Infectious Diseases, Beijing, China

## Abstract

To investigate the prevalence and temporal trend of transmitted drug resistance (TDR), a nationwide cross-sectional survey was conducted among 5627 ART naïve newly diagnosed HIV-infected individuals in 2015 in China. Totally 4704 partial *pol* sequences were obtained. Among them, the most common HIV-1 circulating recombinant form (CRF) or subtype was CRF01_AE (39.0%), followed by CRF07_BC (35.6%), CRF08_BC (8.9%), and subtype B (5.5%). TDR mutations were found in 3.6% of the cases, with 1.1% harboring TDR to protease inhibitors (PIs), 1.3% having TDR to nucleoside reverse transcriptase inhibitors (NRTIs), and 1.6% to non-nucleoside reverse transcriptase inhibitors (NNRTIs). No significant difference was found in the prevalence of TDR, as compared with the results of another nationwide survey performed among ART naïve HIV-infected people in between 2004 and 2005, except in the 16–25 year-old group. In addition, four drug-resistant transmission clusters were identified in phylogenetic trees, accounting for 6.2% (9/145) of the individuals with TDR. Although the rate of TDR remained relatively low in the past 10 years in China, surveillance is still needed to monitor the trend of TDR and to optimize the first-line regimens.

## Introduction

The epidemic of HIV/AIDS has kept rising in China since the first HIV case was reported in 1985. By the end of 2015, there were 557,423 individuals living with HIV/AIDS reported in China, of whom 382,129 were receiving antiretroviral treatment (ART)^1^. The scale-up of ART has led to dramatic reductions in HIV/AIDS-related morbidity and mortality^2–4^. HIV-infected people on suppressive ART have a low risk of onwards HIV transmission^5,6^. However, with the increasing coverage of ART, concerns for the emergence and transmission of HIV drug resistance are arising^5,7–9^.

The rates of transmitted drug resistance (TDR) vary throughout the world. The overall TDR prevalence was estimated to be less than 5% in sub-Saharan Africa and south/southeast Asia, with 2.8% and 2.9%, respectively. However, the estimated TDR rates were rather higher (more than 5%) in other regions, with 5.6% in upper-income Asian countries, 7.6% in Latin America/Caribbean, 9.4% in Europe, and 11.5% in North America^10^. It’s notable that upward trends in TDR continued to be reported in some regions^10,11^. In China, the overall prevalence of TDR among ART-naïve individuals in 2004 and 2005 was 3.8%^12^. The rate of TDR among 16–25 year-old ART-naïve newly diagnosed individuals in China was 3.6% in 2015 (unpublished data), however, there was no recent data on TDR among HIV-infected populations aged above 25 years old.

Since Chinese government officially launched the National Free Antiretroviral Treatment Program (NFATP) in 2003^13^, the number of patients enrolled in this program has increased rapidly year by year. The free ART was estimated to have covered 59% of patients who met the criteria of ART initiation by 2015. During the past years, the first-line regimens had changed a lot as recommended by the national ART guidelines, with Didanosine (ddI) and stavudine (d4T) replaced by lamividine (3TC) and tenofovir (TDF), in 2005 and 2010, respectively. Furthermore, the rate of virological suppression was increased from 78% in 2004 and 2005 to more than 90% in 2013 among Chinese patients on ART. Here, we conducted a nationwide cross-sectional survey in 2015 to characterize TDR among ART-naïve newly diagnosed individuals, and to analyze the trends of TDR over different periods.

## Results

### Characteristics of surveyed populations

A total of 5627 individuals who were diagnosed as HIV positive during Apr. and Jun. 2015 in all the 31 provinces, autonomous regions and municipalities of mainland China were enrolled in this survey. The HIV strains of 4704 (83.6%) participants were successfully sequenced. Of these individuals, the median age was 38 years, 80.0% (3763) were male, and 82.0% (3859) were of Han nationality. More than half of the participants were heterosexuals (53.8%), followed by men who have sex with men (MSMs, 39.4%), and injection drug users (IDUs, 2.4%). According to the results of phylogenetic trees and blasting, the major subtypes and circulating recombinant forms (CRFs) were CRF01_AE (39.0%), CRF07_BC (35.6%), CRF08_BC (8.9%), and subtype B (5.5%). (Table 1).

**Table 1:** Prevalence of TDR by various characteristics between 2004–2005 and 2015.

Characteristics	2004–2005		2015		P Value
	Number	TDR (%)	Number	TDR (%)	
Total	678	26 (3.8)	4704	167 (3.6)	0.71
Age at diagnosis (yrs)					
16–25	51	6 (11.8)	862	28 (3.2)	0.01
26–50	534	16 (3.0)	2749	98 (3.6)	0.51
>50	93	4 (4.3)	1003	39 (3.9)	1.00
Unknown	0	0 (0.0)	90	3 (3.3)	—
HIV risk exposure					
Sexual contact	114	5 (4.4)	4388	161 (3.7)	0.88
Heterosexual	—	—	2533	103 (4.1)	—
MSM	—	—	1855	58 (3.1)	—
IDU	118	4 (3.4)	115	2 (1.7)	0.70
Others or Unknown	456	17 (3.7)	201	4 (2.0)	0.24
Subtype					
01_AE	100	5 (5.0)	1835	73 (4.0)	0.81
07_BC	47	1 (2.1)	1675	47 (2.8)	1.00
08_BC	12	0 (0.0)	418	13 (3.1)	1.00
B	500	18 (3.6)	261	12 (4.6)	0.50
Others	19	2 (10.5)	515	22 (4.3)	0.21

### The prevalence of TDR

**Figure 1:**
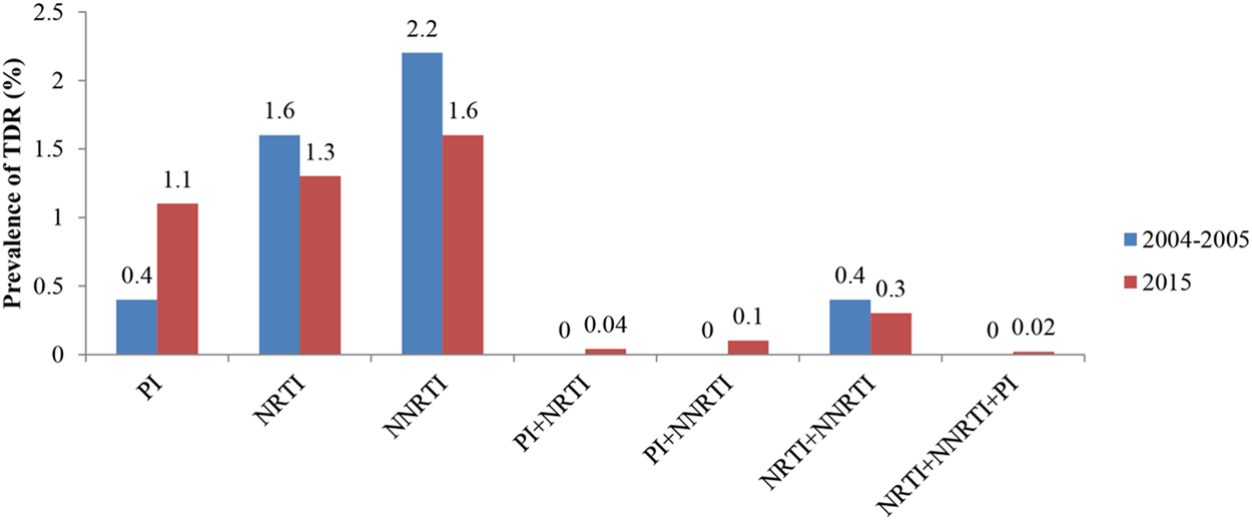
Prevalence of TDR by antiviral drug class between 2004–2005 and 2015.

Among 4704 sequenced individuals, 167 (3.6%) were identified to harbor HIV strains with at least one drug resistance mutation (DRM). The prevalence of TDR to nonnucleoside reverse transcriptase inhibitors (NNRTIs) was 1.6%, followed by TDR to nucleoside reverse transcriptase inhibitors (NRTIs) at 1.3%, and to protease inhibitors (PIs) at 1.1%. The prevalence of dual-class TDR was 0.04% (PI and NRTI), 0.1% (PI and NNRTI), and 0.3% (NRTI and NNRTI). Only one individual (0.02%) was found to harbor triple-class TDR mutations (Fig. 1). The most frequent NNRTI-, NRTI- and PI-related drug resistant mutations (DRMs) were K103N/S, M184V and M46I/L, with the prevalence of 0.6%, 0.3% and 0.6% in the cases, respectively (Table 2).

**Table 2:** HIV drug resistant mutations in 2004–2005 and 2015.

TDR Mutations	2004–2005		2015		P Value
	Number	TDR (%)	Number	TDR (%)	
Total	26	3.8	167	3.6	0.71
PI					
M46I/L	1	0.1	29	0.6	0.21
I85V	0	0	6	0.1	1.00
G73C/S	0	0	4	0.09	1.00
L23I	0	0	3	0.06	1.00
I47V	0	0	2	0.04	1.00
F53L	0	0	2	0.04	1.00
I54L	0	0	1	0.02	1.00
I50V	0	0	1	0.02	1.00
V82L	1	0.1	1	0.02	0.24
N83D	0	0	1	0.02	1.00
I84V	0	0	1	0.02	1.00
N88D	1	0.1	1	0.02	0.24
NRTI					
M184V/I	4	0.6	13	0.3	0.32
T69D/N	0	0	10	0.2	0.47
K219R/Q	2	0.3	9	0.2	0.92
M41L	2	0.3	8	0.2	0.82
L210W	0	0	6	0.1	1.00
T215D/S	3	0.4	6	0.1	0.17
K70Q/R	2	0.3	5	0.1	0.22
K65R	0	0	5	0.1	1.00
D67N/G	3	0.4	5	0.1	0.11
K70R/E	0	0	5	0.1	1.00
V75M	0	0	3	0.06	1.00
Y115F	0	0	1	0.02	1.00
L74I	0	0	1	0.02	1.00
NNRTI					
K103N/S	9	1.3	30	0.6	0.08
V179F	1	0.1	13	0.3	0.83
K101E	3	0.4	13	0.3	0.71
G190A	4	0.6	13	0.3	0.32
V106M/A	1	0.1	12	0.3	0.91
Y181C	1	0.1	7	0.1	1.00
L100I	0	0	1	0.02	1.00
Y188H	0	0	1	0.02	1.00
P225H	0	0	1	0.02	1.00
M230L	0	0	1	0.02	1.00

### TDR in transmission routes, subtypes and age groups

The prevalence of TDR in heterosexuals, MSMs and IDUs was 4.1%, 3.1% and 1.7%, respectively. The prevalence of TDR of CRF01_AE, CRF07_BC, CRF08_BC and subtype B was 4.0%, 2.8%, 3.1% and 4.6%, respectively. There was no significant association between TDR and routes of transmission or subtypes in 2015, and there was no significant difference in TDR rates between the 16–25 year-old group and >25 year-old group (3.2% vs 3.6%, P = 0.48).

### Comparison of TDR among different study periods

The rates of TDR in this study were compared with the results from another nationwide survey, carried out in 2004–2005 among ART naïve HIV-infected individuals. There was no significant difference in the rates of TDR among different routes of transmission or subtypes between the two study periods. However, the rate of TDR in the 16–25 year-old group was significantly higher in 2004–2005 (P = 0.01, Table 1). M184V was the most frequent NRTI-related mutation during 2004–2005, and the most frequent NNRTI-related mutation was K103N/S. PI-related mutations were rare and only found in 3 individuals. No significant difference was found in the prevalence of specific mutations between the two surveys (Table 2).

### Phylogenetic analyses of the main subtypes

**Figure 2:**
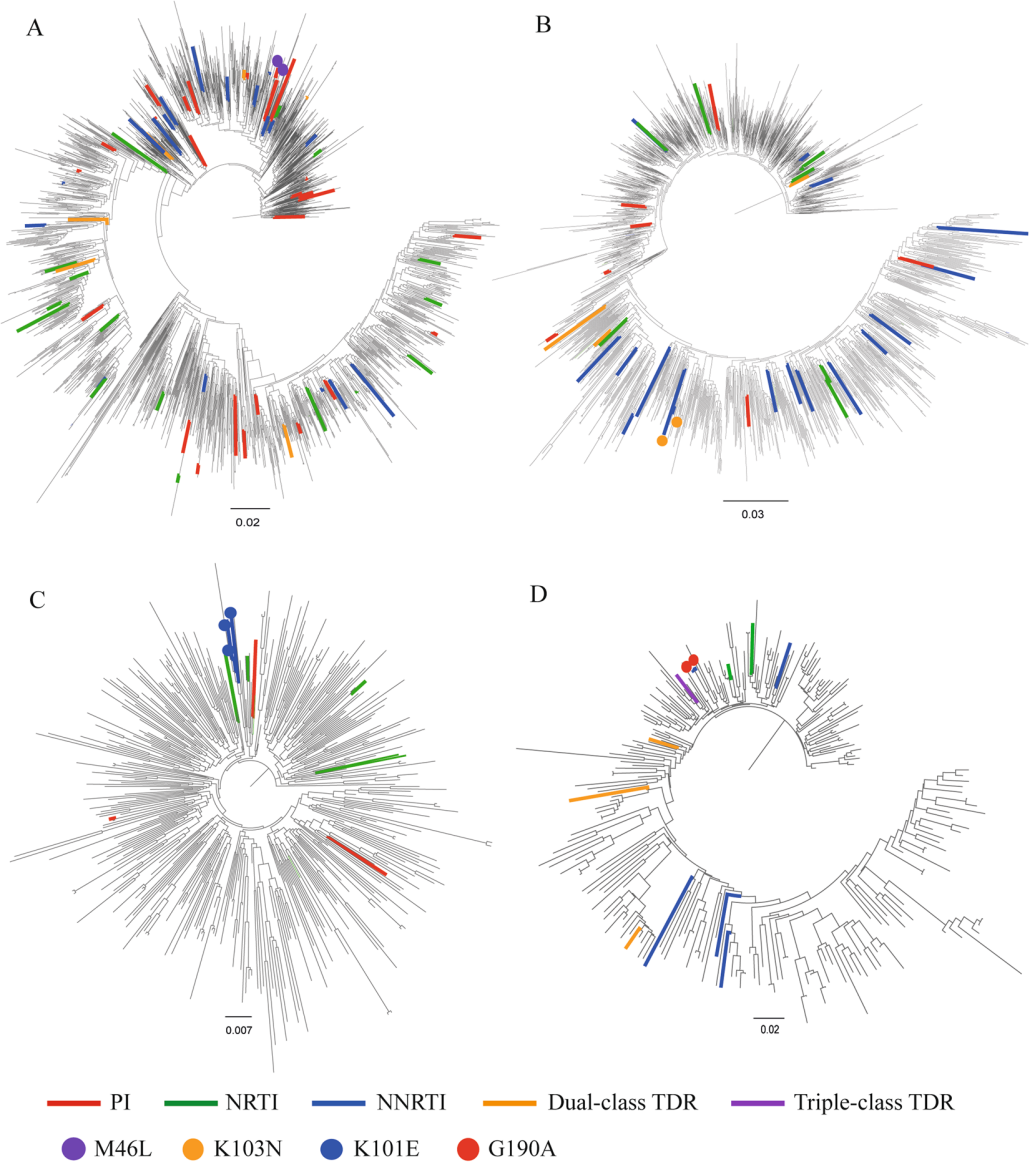
The maximum likelihood phylogenetic trees of 4198 main subtype sequences were constructed using GTR + Gamma substitution model in Fasttree. (**A**) phylogenetic tree of 1835 CRF 01_AE sequences. (**B**) phylogenetic tree of 1675 CRF 07_BC sequences. (**C**) phylogenetic tree of 418 CRF 08_BC sequences. (**D**) phylogenetic tree of 261 subtype B sequences. Nine sequences of the 4 drug-resistant transmission clusters are marked with different color dots.

To explore the possible transmission relationship between individuals harboring drug-resistant strains, four phylogenetic trees were constructed based on sequences belonging to CRF01_AE (1835), CRF07_BC (1675), CRF08_BC (418), and subtype B (261), respectively (Fig. 2). Four drug-resistant transmission clusters including 9 sequences were identified in four phylogenetic trees. The DRMs in the clusters were M46L (cluster A), K103N (cluster B), K101E (cluster C), and G190A (cluster D). The presence of drug-resistant strains within drug-resistant transmission clusters accounted for 6.2% (9/145) of the total drug-resistant strains in this study.

### Drug resistance-associated transmission network analysis

To explore the effect of TDR on viral transmission based on the transmission network, the 4704 sequences were divided into two groups: drug-resistant (167) and drug-sensitive (4537). The overall rate of clustering was 68.7%. The rate of clustering in drug-resistant strains was 60.5%, lower than in drug-sensitive strains (69.0%), P = 0.02. In addition, the degree was significantly lower in drug-resistant strains (P < 0.0003, Table 3).

**Table 3:** Degree comparison for drug-resistant and drug-sensitive individuals in 2015.

Drug resistance situation	Number	Inter-quartile range	Median	P Value
Total	4704	7	2	
Yes	167	3	1	
No	4537	8	2	<0.0003

## Discussion

In this study, we investigated TDR among ART-naïve HIV-infected individuals in China who were newly diagnosed in 2015. The overall prevalence of TDR was 3.6%. Except a significant decreasing trend of TDR in the 16–25 year-old group, there was no significant difference in the rates of TDR in the whole study populations, or in any specific transmission routes and subtypes between the surveys conducted in 2004–2005 and 2015. In general, the prevalence of TDR remained low in China. This prevalence was comparable to other recent surveys in South America, South Africa and Asia, which showed 3.8% TDR in 2012 in Maranhão State, Northeast Brazil^14^, 4.7% in 2011 in rural KwaZulu-Natal, South Africa^15^, 4.8% during 1999 and 2012 in South Korea^16^, and 4.0% during 2008 and 2010 in Thailand^17^. In contrast, the prevalence of TDR in Europe and North America was much higher. A study carried out among HIV-infected individuals from 26 countries in Europe who were newly diagnosed between 2008 and 2010 revealed that the overall prevalence of TDR was 8.3%^18^. A sensitive sentinel mutation screening revealed that the rate of TDR in the United States was 13.6% during 2009 and 2011^19^. Even higher rate of TDR was 22.5%, found in Metropolitan Washington, DC during 1994 and 2013^20^. It is not surprising those countries like China has a lower rate of TDR, as the duration of access to ART was much shorter. In addition, HAART was applied from the beginning of the NFATP in China, as opposed to the situation in Europe and North America which experienced an extended period of much less effective regimens^21^. In 1987, zidovudine (AZT) was approved by US FDA (Food and Drug Administration) for use in patients with advanced HIV. Combination NRTI therapy was proven more efficacious in 1995. Triple drugs regimens were suggested to use during the Vancouver AIDS Conference in 1996 by IAS-USA (International Antiviral Society–USA)^21^.

The main transmitted DRMs were different between China and countries in Europe and North America. The most frequent NRTI-associated DRM found in our study was M184V, whereas were T215rev (revertant mutation) and M41L in Europe and North America^18–20,22–28^. The most frequent PI- associated mutation in our study was M46I/L, which is related to the high prevalence of CRF01_AE in China^29^. The most frequent PI-associated mutations were L90M and M46I/L in Europe and North America^18–20,22–28^. Consistent with that of Europe and North America, the most frequent NNRTI-associated DRM in China was K103N/S. The differences of the main types of DRM can be explained by the different ART regimens used and subtype distribution between regions. Firstly, zidovudine (AZT), lamivudine (3TC), tenofovir (TDF), abacavir (ABC), efavirenz (EFV), nevirapine (NVP), ritonavir-boosted lopinavir (LPV/r) were provided through the NFATP in China. Not all PIs are available and none of the integrase strand transfer inhibitors (INSTIs) are provided in China compared to Europe and North America. Secondly, the major subtypes in China are CRF01_AE and CRF07_BC, whereas it is subtype B in Europe and North America.

Four clusters containing HIV strains sharing the same DRM were found in the present study. The presence of drug-resistant strains within transmission clusters accounted for only 6.2% (9/145). This revealed that the prevalence of TDR was not concentrated in study populations. This finding is consistent with the low prevalence of TDR in China.

The degree and rate of clustering in transmission networks were significantly lower in drug-resistant strains. This may be explained by the lower replication capacity of the resistant virus. The resistant virus was transmitted only approximately 20% as frequently as expected according to a previous study^30^.

As with other observational studies, our study has limitations. Firstly, a proportion of the studied individuals might not be recently infected, and the drug-resistant strains in plasma become minor quasi-species after a period of infection. The Sanger sequencing method may underestimate the prevalence of TDR. Secondly, there would be a biased sampling since only 10% of individuals diagnosed in the second quarter of 2015 were randomly enrolled in this study.

In conclusion, the overall prevalence of TDR among recently diagnosed individuals in China remained at a low level in the recent 10 years. The prevalence of TDR was not concentrated in 2015. We suggest that effective measures are still needed to strengthen monitoring and guide ART usage.

## Methods

### Study population

We conducted a cross-sectional survey in 31 provinces, autonomous regions and municipalities of mainland China. Inclusion criteria for the subjects (1) were over 16 years old, (2) were permanent residents, (3) were diagnosed as HIV seropositive from April 2015 to June 2015, and (4) were ART naïve. Individuals who met the criteria were stratified by random sampling. The sampling ratio of each province was determined by the average number of HIV-infected individuals reported in 2011–2013. For provinces with fewer reported cases, higher sampling ratios were used to assure statistical confidence. The sampling ratios for provinces with >2000, 1200–2000, 800–1200, 250–800, and <250 cases were 5%, 10%, 12.5%, 15% and 20%, respectively. All patients provided written informed consent for participation in this study.

To explore the changes of TDR in the recent 10-year period in China, the data of a cross-sectional survey conducted in 28 provinces from September 2004 to October 2005 was introduced into the analysis. The data contained sequences and basic information of 676 ART-naïve individuals^12^.

### Identification of transmitted drug resistance

Whole blood samples were collected at local Centers for Disease Control and Prevention (CDCs). For plasma samples, which were separated by centrifugation, HIV drug resistant test was carried out. The HIV *pol* region (protease 1–99 amino acids and reverse transcriptase 1–250 amino acids) was amplified by an in-house polymerase chain reaction protocol^31^. The nucleotide sequences for the *pol* region were assembled and edited in Sequencher version 4.10 and BioEdit version 7.0.9.1. Transmitted drug resistant mutations were identified using CPR 6.0 in the Stanford HIV Drug Resistance Database (https://hivdb.stanford.edu/).

### HIV subtyping

For HIV subtyping, the edited sequences were aligned with reference sequences on HIV databases (http://www.hiv.lanl.gov/content/index). Phylogenetic trees were constructed using a neighbor-joining method with 1000 bootstrap replicates with MEGA6.0. The HIV subtypes and circulating recombinant forms (CRFs) were identified with a bootstrap equal to or above 70%. The results were checked using HIV Blast on HIV databases.

### Phylogenetic analyses

To avoid the effect of convergent evolution, 43 codons for drug resistance surveillance were removed from all of the aligned sequences. The total length of the sequences was 910 bp. Four phylogenetic trees were respectively constructed based on the main circulating recombinant forms (CRFs) and subtypes predominating in China: CRF01_AE, CRF07_BC, CRF08_BC, and subtype B. Phylogenetic relationships were estimated using a maximum likelihood approach with a General Time Reversible + Gamma (GTR + η) model and 1000 replicates bootstrap in Fasttree. The criteria for identifying drug-resistant transmission clusters: (1) with a bootstrap above 90% and (2) at least two individuals in the same cluster carrying the same DRMs. These trees were edited and visualized with FigTree 1.4.3 and Adobe Illustrator CC 2014.

### Transmission network analysis

For constructing the transmission network, genetic distances were first calculated using the Tamura-Nei 93 nucleotide substitution model (TN93) with HYPHY version 2.2.4. A putative transmission linkage was inferred if the genetic distance between two sequences was below 1.5%. This cutoff threshold was based on an evolutionary rate of 0.7%/year for HIV-1 pol within individuals^32–34^. The transmission network was then constructed and analyzed with Cytoscape version 3.2.0. The rate of clustering was defined as the percentage of individuals segregated into the network. The degree of each individual in the network was defined as the number of links with other individuals.

### Statistical analyses

Categorical variables were compared using the χ^2^ test, Fisher exact tests, or logistic regression analysis. The statistical significance was defined as P < 0.05. All statistical analyses were performed using SAS version 9.3 (SAS Institute, Cary, NC).

### Ethics statement

Institutional review board (IRB) approval was granted by National Center for AIDS/STD Control and Prevention (NCAIDS), Chinese Center for Disease Control and Prevention (China CDC). All experimental protocols were approved by IRB at NCAIDS, China CDC, according to the international and Chinese ethical guidelines, and the methods were carried out in accordance with the approved guidelines. All patients were willing to provide informed consent for this research.
